# Observations on Hydrocephalus, with a Case

**Published:** 1815-09

**Authors:** G. D. Yeats

**Affiliations:** King-Street, St. James's


*For the London Medical and Physical Journal.*

### *Observations on Hydrocephalus, with a Case;* by G. D. Yeats, M.D.

HAD not experience fully convinced me of the great importance and necessity of attending with watchful care to the derangement of the abdominal viscera in children, on account of the fatal consequences which too frequently result, I should not have requested the insertion of the following instructive case in your valuable Journal. The most dangerous consequence of such morbid action is that disease of the head producing water in the brain; and the present case is one among many which I have witnessed of the accession of disease within the cranium, rapidly succeeding to, and accompanying derangement of the digestive organs. I wish particularly to direct the attention to the history of the symptoms previous to the convulsive attack, and to the lightning-like manner in which the transfer, or rather the co-existence of disease, with that of the chylopoietic viscera, took place in the brain, and the rapidity with which it run its fatal course. In these previous symptoms, the derangement in the functions of the abdominal viscera is particularly distinguishable, and especially the puffiness in the epigastric region, which, in my letter to Dr. Wall on Hydrocephalus, I have endeavoured to explain as a discriminating and early symptom of this complaint, and as originating from a morbid condition of the duodenum.* In many cases

---

* A statement of the early symptoms which lead to the disease termed water in the brain, p. 41.

so extremely rapid and unexpected is the attack upon the brain, that the inexperienced in the early symptoms of the disease are in the midst of the greatest difficulties and dangers before they are aware of the presence of any other than trifling ailments. In the present instance, so rapid was the progress, that the child was suddenly seized with the fatal effects of irritation on the brain at noon on the Monday, and died on the Thursday following, within seventy-two hours of the convulsive seizure; not, however, without that warning as exhibited in the morbid condition of the digestive organs; an early attention to which, on the principle that they were the precursors of the disease in the brain, had the friends been aware of the circumstance, would probably have prevented the fatal termination. The practice pursued was active, it consisted in bleeding with leeches freely from the head, and in the use of the requisite purgatives, calculated to promote the regular peristaltic motion and evacuation of the intestinal canal. Blisters were not overlooked, and the use of the mercurial ointment had been commenced. I subjoin to the case the dimensions of an hydrocephalitic head, compared with the dimensions of the head of a healthy child of the same age and sex, as a matter of curiosity.

J. H. a very lively child, fourteen months old, was suddenly seized (April 24) with general convulsions, about twelve o'clock at noon, which lasted one hour, when they remitted, but returned with considerable violence at two; the convulsions were now almost entirely confined to the right side, the face was drawn to that side, and the head turned over the right shoulder, with convulsive catches in the upper and lower extremities, similar to the quick spasmodic contractions produced by galvanism in frogs: froth issued from the mouth; insensibility prevailed; the pupils were dilated; strabismus took place, alternating with rolling of the eyes. I saw her in this state. The countenance was flushed and puffy; the pulse very rapid and hard; there was considerable heat over the whole surface of the body. The *epigastric region was turned with a tensiveness over the abdomen.* The convulsions subsided into a stupor, which remained more or less the whole evening. On entering the room the next morning, the child was lying in bed sawing the air with her left hand. I immediately knew, from this symptom, that the right side was paralytic; it proved so upon examination; a stupor was present, the eye-lids were nearly closed, on opening them the eyes were observed to be turned up; the pupils were contracted; she took greedily during the night a pint of arrow-root. 4 p. m. The pupils contract by light, the eyes roll about and occasionally remain main fixed for a while in the internal canthi; the child is apparently sensible; the bowels answer, but not fully; the pulse is quick, but not irregular; face pale, skin sufficiently warm; sickness is at times troublesome. 9 at night, lies sleeping in a stupid state with slow breathing. Pulse very quick, but regular; eye-lids closed; upon examination, pupils contract to a very small point; left eye turned outwards; has been frequently sick and vomited; no motion from the bowels, nor water passed since 4 o'clock; paralytic affection of the right side remains, and the fingers of the right hand are spasmodically closed.

26th, eleven a. m. Has past a quiet night, only sick once; bowels kept open by the powders composed of calomel, scammony, and aloes; stools watery, and colouring the napkins of a green colour; has past urine; pupils contract healthily; still saws with the left arm; has taken breast-milk and arrow-root.

4 p. m. Both eyes, with dilated pupils, are turned to the right side; the right side is still paralytic, and the fingers spasmodically contracted, constantly sawing with the left arm; no evacuation since the morning; before leaving the room, the eyes came to their natural position, with contracted pupils.

27th, eleven a. m. Passed a restless night; right side continues perfectly paralytic; is constantly sawing with the left arm; hands cold and of a purple colour; pupils much dilated; eyes open and fixed; is insensible; the breathing is quick, with occasional yawning; rattling in the throat; is moribund; died at 12 o'clock at noon.

Although every influence was used, permission to open the body could not be obtained, which I very greatly regretted.

*Previous to the convulsive attack, the child, of a very lively active disposition, was observed to droop; the bowels became unusually costive; the flesh put on a more flabby feel; the child's usual good spirits declined. On Saturday night, two days previous to the convulsions, it appeared to be in great pain by crying much, a circumstance very unusual with the child; was very feverish, with flushed checks, often changing colour; was more inclined to remain quiet and at rest in its mother's arms; was occasionally sick, but did not vomit; a cough was troublesome, and which was sometimes so violent as to end in vomiting, bringing up only phlegm; was unusually thirsty. It was noticed for the first time, on the morning of the day on which the convulsions occurred, that the light was troublesome to the eyes. About a week before this fatal seizure, the costive state of the bowels run into a diarrhœa of so relaxed a nature, that astrin-*

 *gents*

*gents were resorted to.* There was no symptom of irritation from teething.

In passing through Newport Pagnell, in Bucks, on July 26th last, to visit a patient in that county, Mr. Collison, an intelligent and attentive surgeon there, brought me a child about six months' old, with a very enlarged head, from water collected within it. The child was born with spina bifida; when two months old the watery protuberance at the bottom of the spine disappeared, when the head began to enlarge, and gradually increased to its present unwieldy bulk. The following are the comparative dimensions between this child's head and a healthy one.

| *Diseased Head.* | Inches. | *Sound Head.* | Inches. |
|---|---|---|---|
| Across the head from tip to tip of the ears .... | 16¾ | Ditto.............. | 8¾ |
| From between the eye-brows, across the top of the head, along the sagittal suture to the tuberosities of the occiput ............... | 18¾ | Ditto .............. | 10¾ |
| Circumference of the head round the temples, forehead, and occiput | 25¾ | Ditto .............. | 16½ |

*King-street, St. James's;*

*August* 1, 1815.

G. D. YEATS, M.D.

